# Supplementary material for: Splice-Junction-Based Mapping of Alternative Isoforms in the Human Proteome
Source: Cell Rep. Author manuscript; Available in PMC 2020 Jan 15. (PMC6961840; doi:10.1016/j.celrep.2019.11.026)
Supplement: 3 [file NIHMS1546469-supplement-3.zip › DF2/PXD0010154/Colon-36-Q96MT8-LELGLHECSLPVSPLGSIATR.pdf]

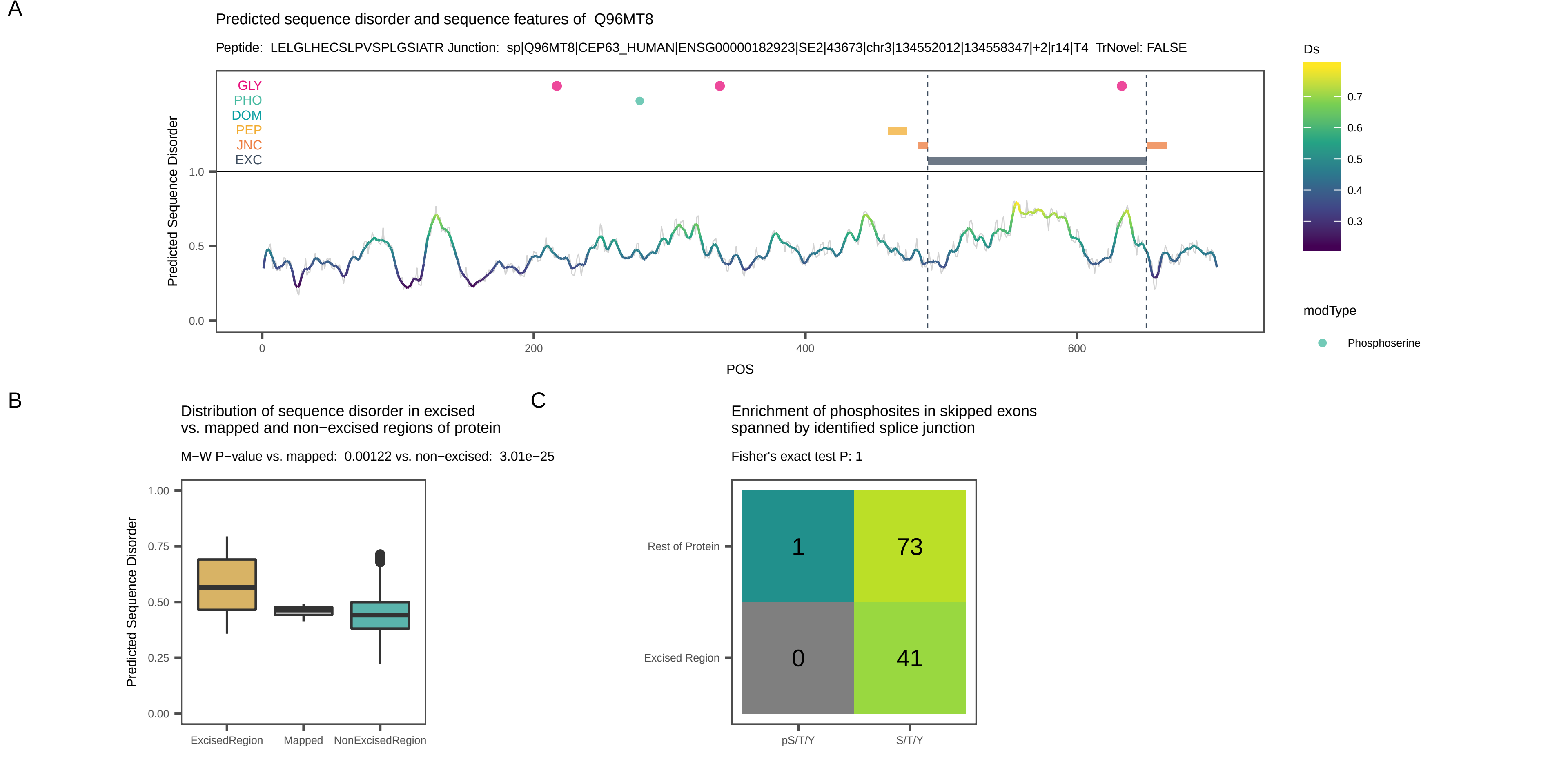

A
Predicted sequence disorder and sequence features of Q96MT8
Peptide: LELGLHECSLPVSPLGSIATR Junction: sp|Q96MT8|CEP63_HUMAN|ENSG00000182923|SE2|43673|chr3|134552012|134558347|+2|r14|T4 TrNovel: FALSE
GLY
PHO
DOM
PEP
JNC
EXC
Predicted Sequence Disorder
1.0
0.5
0.0
0
200
400
600
POS
Ds
0.7
0.6
0.5
0.4
0.3
modType
Phosphoserine
B
Distribution of sequence disorder in excised
vs. mapped and non−excised regions of protein
M−W P−value vs. mapped: 0.00122 vs. non−excised: 3.01e−25
Predicted Sequence Disorder
1.00
0.75
0.50
0.25
0.00
ExcisedRegion
Mapped
NonExcisedRegion
C
Enrichment of phosphosites in skipped exons
spanned by identified splice junction
Fisher's exact test P: 1
Rest of Protein
Excised Region
1
73
0
41
pS/T/Y
S/T/Y
